# Hospital doctor turnover and retention: a systematic review and new research pathway

**DOI:** 10.1108/JHOM-04-2023-0129

**Published:** 2024-02-27

**Authors:** Siva Shaangari Seathu Raman, Anthony McDonnell, Matthias Beck

**Affiliations:** Cork University Business School, University College Cork, Cork, Ireland

**Keywords:** Employee turnover, Retention, Embeddedness, Hospital doctor

## Abstract

**Purpose:**

Society is critically dependent on an adequate supply of hospital doctors to ensure optimal health care. Voluntary turnover amongst hospital doctors is, however, an increasing problem for hospitals. The aim of this study was to. systematically review the extant academic literature to obtain a comprehensive understanding of the current knowledge base on hospital doctor turnover and retention. In addition to this, we synthesise the most common methodological approaches used before then offering an agenda to guide future research.

**Design/methodology/approach:**

Adopting the PRISMA methodology, we conducted a systematic literature search of four databases, namely CINAHL, MEDLINE, PsycINFO and Web of Science.

**Findings:**

We identified 51 papers that empirically examined hospital doctor turnover and retention. Most of these papers were quantitative, cross-sectional studies focussed on meso-level predictors of doctor turnover.

**Research limitations/implications:**

Selection criteria concentrated on doctors who worked in hospitals, which limited knowledge of one area of the healthcare environment. The review could disregard relevant articles, such as those that discuss the turnover and retention of doctors in other specialities, including general practitioners. Additionally, being limited to peer-reviewed published journals eliminates grey literature such as dissertations, reports and case studies, which may bring impactful results.

**Practical implications:**

Globally, hospital doctor turnover is a prevalent issue that is influenced by a variety of factors. However, a lack of focus on doctors who remain in their job hinders a comprehensive understanding of the issue. Conducting “stay interviews” with doctors could provide valuable insight into what motivates them to remain and what could be done to enhance their work conditions. In addition, hospital management and recruiters should consider aspects of job embeddedness that occur outside of the workplace, such as facilitating connections outside of work. By resolving these concerns, hospitals can retain physicians more effectively and enhance their overall retention efforts.

**Social implications:**

Focussing on the reasons why employees remain with an organisation can have significant social repercussions. When organisations invest in gaining an understanding of what motivates their employees to stay in the job, they are better able to establish a positive work environment that likely to promote employee well-being and job satisfaction. This can result in enhanced job performance, increased productivity and higher employee retention rates, all of which are advantageous to the organisation and its employees.

**Originality/value:**

The review concludes that there has been little consideration of the retention, as opposed to the turnover, of hospital doctors. We argue that more expansive methodological approaches would be useful, with more qualitative approaches likely to be particularly useful. We also call on future researchers to consider focussing further on why doctors remain in posts when so many are leaving.

## Introduction

Hospital doctors are the backbone of any health organisation and public health system (Keogh, 2013). They are at the epicentre of public access to health services and a key determinant in the quality of care for all (Oliveri *et al.*, 2004). Therefore, a healthy society requires an appropriate number of qualified hospital doctors. However, concerns are growing about the increasing shortages of hospital doctors (World Health Organization WHO, 2022). Research indicates that the voluntary turnover of hospital doctors is problematic in many countries and rising (Waldman, 2010). For example, in 2019 the turnover of hospital physicians in the US was reported as almost 11% (Pappas *et al.*, 2022). Similarly, in the UK, there continues to be a year-on-year decline in doctors undertaking hospital training programmes, while the number of doctors leaving the NHS is increasing (Wilson *et al.*, 2021; Lock and Carrieri, 2022). In Ireland, around 2000 medical doctors voluntarily departed from the Irish Medical Register in 2018, reflecting a 9% exit rate (Malone, 2020).

This turnover can negatively affect staff morale and the health of doctors who remain, whilst insufficient staffing has been shown to increase the incidences of medical errors (Kirch and Petelle, 2017; Chojnicki and Moullan, 2018). The costs of recruiting and training new doctors are also substantial given the highly skilled nature of the work (Fibuch and Ahmed, 2015). High turnover also brings substantial intangible costs in terms of the loss of organisational and tacit knowledge. Most critically, appropriate staffing levels, contribute significantly to the provision of quality patient care and enhances the continuity of care within the health service (Mok *et al.*, 2020).

Several reviews have been conducted on the turnover of medical doctors. These have, however, typically encompassed several types of doctors, including general practitioners, those from hospitals but within specific specialities, and also other medical professionals, such as nurses (e.g. Lichtenstein, 1984; Misra-Hebert *et al.*, 2004; Poon *et al.*, 2022; de Vries *et al.*, 2023). Systematic reviews of research dedicated to hospital doctor turnover have been less evident. We argue that this is an issue that needs redressing because hospital doctors have substantially different responsibilities and/or work contexts vis-à-vis general practitioners, nurses, and other healthcare professionals (Khan *et al.*, 2018). Therefore, conclusions are difficult to be drawn towards this category of doctor. In addition, hospital doctors are an especially important group of influence within hospital workplaces as they hold positions of clinical leadership and responsibility at the front-line of patient care (Godlee, 2008).

The objective of this paper is to critically review and synthesise the current body of research associated with hospital doctor turnover and retention. Our overarching guiding research question we address is what does research tell us about why hospital doctors leave? We focus on two further sub-research questions namely, what are the most common methodological approaches employed by research in this domain, and what are the key knowledge gaps around hospital doctor turnover? Following on from this, we seek to advance knowledge by proposing an agenda to guide future research.

## Theory

Since the pioneering work of March and Simon (1958) our understanding of employee turnover has expanded considerably. Five different paths have been identified which are seen as especially relevant to explaining employee turnover and which dominate the extant literature (Lee and Mitchell, 1991, 1994; Lee and Maurer, 1997; Mitchell *et al.*, 2001). The first three paths are centred on the influence of *shocks* which causes an employee to review their current job position. The first involves a *shock* which is typically non-job related. For example, an employee might set themselves a target of saving money, and, once this goal is achieved, they may decide to resign. The second and third pathways relate to unanticipated *shocks*, where negative or positive external triggers motivate an employee to leave. The second pathway could involve a situation where an employee receives a poor or negative performance review from a manager that prompts the individual to resign. The third path may involve a situation where, for example, a recruitment agency contacts an individual, prompting them to consider a change and pulling them away from their current job. The final two paths centre on employee dissatisfaction, which may or may not lead the individual to depart for another role. All pathways have been shown as empirically relevant and turnover-induced, although different types of shocks have, on occasion, been shown to be of greater relevance than employee dissatisfaction (Holtom *et al.*, 2005; Kulik *et al.*, 2012).

Another common conceptual classification of behaviour related to employee turnover is the macro, micro, and meso lenses (Bolibar, 2016). The macro-level lens is commonly employed by economists in analysing the role of market forces in employee turnover (Banerjee and Gaston, 2004). The micro factor, which centres on the individual or psychological level, typically focusses on the relationship between job dissatisfaction and organisational commitment to turnover. Focussing on the space between these levels, the meso-level lens considers organisational factors and how they influence turnover with importance given to the social context in which individuals are situated. The micro level and the importance of individual attitudes in people’s decisions to leave their jobs has been the most dominant approach in the wider employee turnover literature (Pfeffer, 2001).

In recent decades, scholars interested in employee turnover have recognised the merits of turning the why employees leave question on its head, and asking why employees choose to stay? This focus has been heavily centred on the development of job embeddedness theory (Mitchell *et al.*, 2001) which incorporates three dimensions. *Fit* describes the extent to which a person’s skills, interests, and values align with their work and the organisation; *links* are the formal and informal connections that exist between people; and, *sacrifice* reflects the real and perceived costs that an individual may ascribe to leaving their job. The basic principle is that, the more embedded an employee is the less likely it will be that they will leave voluntarily.

## Methods

**Figure 1:**
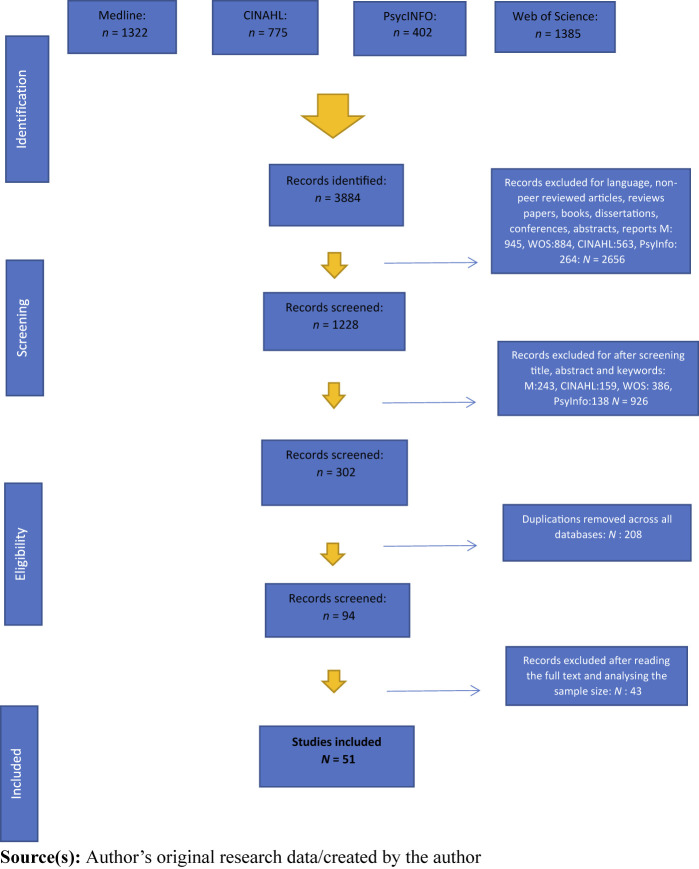
Overview of systematic literature review search process

The Preferred Reporting Items for Systematic Reviews and Meta-Analyses Protocols (PRISMA) were adopted to conduct this review (see Figure 1). We utilised the CINAHL, MEDLINE, PsycInfo, and Web of Science databases because they provide strong coverage of the medical context, and subject matter, and they are the most likely to contain multidisciplinary articles relevant to our objective. Our search strategy had to be tailored due to the way that the different databases operate. Searches in CINAHL, MEDLINE and PsycInfo were conducted using identical search strings: “voluntary turnover” OR turnover OR “intention to leave” OR “intention to quit” OR “consider leaving” OR retention OR “intention to stay” AND “hospital” AND “doctor” OR “physician” OR “consultant”. Meanwhile, for the Web of Science review, wildcard (*) searches incorporated “voluntary turnover” OR “turnover” OR “intention to leave*” OR “intention to quit*” OR “consider leave*” OR “retention” OR “intention to stay*” AND “hospital*” AND “doctor*” OR “physician*” OR “consultant*”. These search strings were developed after substantial sensitivity analyses had been conducted, which involved testing combinations of keywords and sequences in each database to identify the most appropriate search terms that captured the most relevant results in line with our objective (Bramer *et al.*, 2018). We decided against employing a starting date for the review as we were keen to evaluate the evolution of this research longitudinally.

### Eligibility criteria

The review centred on empirical research papers that focussed on hospital turnover and retention. For a paper to be included in our results, the following criteria had to be met: (1) it had to published in a peer-reviewed academic journal; (2) it had to be written in English; (3) it had to focus on medical doctors in a hospital setting; and (4) it had to contain at least one of the search terms in the title or abstract or keywords. Review papers, books, conference proceedings, and news articles were therefore excluded. Papers focussing solely on the turnover and retention of other types of healthcare personnel (e.g. family physicians, general practitioners, nurses, midwives, pharmacists, and lab assistants) were excluded. A total of 3,884 papers were initially identified through the database search. Subsequently, in a two-step process where the above criteria were applied, we were left with 302 papers. An additional 208 were then removed due to duplication. At this juncture, we assessed the records for eligibility based on the content of each paper. After reading each paper in detail to determine relevance, a further 43 papers were excluded. This left the final sample at 51 papers.

### Data extraction and analysis

A template was created, using a Microsoft Excel spreadsheet, to classify data extracted from each paper. This spreadsheet recorded detailed information within each paper which included the name of the author(s), year of publication, the location(s) of the empirical data collection, paper title, paper aim and objectives, the theories used/applied (if any) in each paper, keywords, methodological details (i.e. method, sample group and sizes, demographics, level of analysis, key findings, and future research suggestions. We then undertook a thematic analysis of the findings (Clarke and Braun, 2017). This initially involved an open coding approach (i.e. without a pre-set code), before we then assigned to the aforementioned macro, micro, and meso framework. Some articles covered more than one level, and in these cases, we report across macro, micro and meso levels as appropriate.

## Results

### Descriptive results

**Figure 2:**
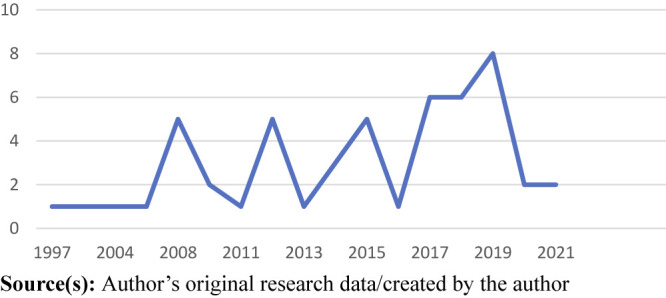
Number of articles published per year

The first empirical paper was published in 1997 demonstrating the relative recency of this stream of research on hospital doctors. It was not until the late 2000s that there was an exponential increase in research. Specifically, 48 of the 51 papers appeared between 2008 and the peak year - 2019 (see Figure 2). Multi-authored papers were common (*n* = 49), and primary publication outlets tended to be medical or healthcare-focussed journals (*n* = 38) rather than HR and work psychology type journals which much of the wider employee turnover literature has been published. These articles were spread across 35 different journals with *BMC Health Services Research* the most popular accounting for 8 articles (see Table 1).

### Macro level factors

There were 12 studies that specifically considered macro factors and their relationship with hospital doctor turnover. Economics and politics emerged as impinging factors that, in some cases, created less than positive conditions for doctors. Specifically, research scrutinised the role and influence of regulations, which some doctors saw as distorting the aims of the profession and damaging their working conditions and healthcare environment. The lack of government funding and/or limited healthcare expenditure were seen to be severely impacting the provision of resources, such as hospital beds and subsidies per bed, along with reduced per capita funding for personnel (Zhang *et al.*, 2019; Brugha *et al.*, 2021). For example, Gutacker *et al.* (2019) noted that, while UK government initiatives, such as the medical revalidation system, provided greater assurance to patients and the public, they were having a negative impact on doctors and were partly responsible for increased turnover.

Institutional issues such as poorly defined governance structures, poor work policies (affecting the long-term availability of jobs for hospital doctors, including permanent and consultant positions), uncertainty about future work contracts, and rural placements, together with limited funding and limited placement opportunities for postgraduate training were all reported as contributing to increased doctor turnover in several jurisdictions (Sharma *et al.*, 2012; Zhang *et al.*, 2017; Humphries *et al.*, 2018; Lambert *et al.*, 2018; Smith *et al.*, 2018; Brugha *et al.*, 2021; Byrne *et al.*, 2021). For example, researchers noted that the health system in Ireland has not recovered sufficiently since the austerity period arising from the 2008 global financial crisis. This has impacted hospital doctors, who have worked in a highly stretched and understaffed health system which, in turn, has led to Irish hospitals being increasingly viewed as undesirable places to work (Humphries *et al.*, 2018).

Nationality and linked visa requirements have also been shown to influence doctor turnover. When doctors require a visa/work permit, this can lead to significant difficulties in obtaining clearance on the initial application, and at the renewal stages which can be a source of real challenge (Smith *et al.*, 2018). Moreover, nationality can influence doctors’ eligibility for government postgraduate training courses and other related schemes (Bruce-Brand *et al.*, 2012). Being ineligible or unsuitable for training programmes obstructs a doctor’s career pathway to becoming a consultant, and this factor has been identified as a contributor to hospital doctor turnover. Countries that viewed to be more straightforward and timely visa processing systems (e.g. Australia and New Zealand) appear to benefit over countries with more complex, cumbersome systems (Smith *et al.*, 2018) when it comes to doctor turnover and retention.

### Micro level factors

a) Individual attitudes

Job satisfaction was the most common factor that researchers focussed on and it was consistently found to be an important explanatory variable behind hospital doctor turnover. Some 35 studies reported that individuals experiencing low job satisfaction led to increased turnover (Pathman *et al.*, 2002; Fuß *et al.*, 2008; Williams *et al.*, 2010; Bruce-Brand *et al.*, 2012; Sharma *et al.*, 2012; Heponiemi *et al.*, 2014; Ali Jadoo *et al.*, 2015; Tsai *et al.*, 2016; Roy *et al.*, 2017; Pantenburg *et al.*, 2018; Zhang *et al.*, 2019). Most studies examined the direct relationship between job satisfaction and turnover, but some evaluated the impact of work-related stress and burnout on these issues. For example, high levels of stress and workplace burnout were associated with lower satisfaction which in turn increased turnover (Pathman *et al.*, 2002; Fuß *et al.*, 2008; Zhang and Feng, 2011; Zhang *et al.*, 2017). Similarly, high job demands and individual difficulties in coping have been shown to lead to burnout, reduced job satisfaction, and increased withdrawal behaviours (Zhang and Feng, 2011; Tziner *et al.*, 2015; Roy *et al.*, 2017). Low job satisfaction has regularly been shown to result from excessive working hours (Ochsmann, 2012; Tsai *et al.*, 2016); workplace violence; and bullying and low job control (Heponiemi *et al.*, 2014); as well as insufficient work-life balance; poor relationships at work; lack of training opportunities; reduced work enjoyment, income, and safety (Bruce-Brand *et al.*, 2012; Ali Jadoo *et al.*, 2015; Pantenburg *et al.*, 2018); work and family interfering with each other (Fuß *et al.*, 2008); and low job autonomy (Degen *et al.*, 2014).

The data suggested that doctors’ low levels of job satisfaction did not necessarily relate to their views of patient care or of their overarching career paths (Pathman *et al.*, 2002; Wang *et al.*, 2015; Pantenburg *et al.*, 2018). In fact, many doctors were found to be highly satisfied with their relationship with patients (patient care), and their careers, due to the meaningfulness of their jobs, intrinsic interest, level of challenge, self-fulfilment, and the social status received. High levels of stress, burnout, and frustration relating to aspects of hospital doctors’ work were also shown to result in higher rates of turnover. Similarly, deteriorating mental health, depression, and high levels of anxiety (Hamidi *et al.*, 2018) sleep deprivation, poor health, and well-being (Tziner *et al.*, 2015) were shown as significant explanatory variables.

On the other hand, strong professional commitment (i.e. commitment towards the medical profession) was shown to bring higher levels of job satisfaction which increased the intention to stay (Lachman and Noy, 1997). Studies that focussed on intention to stay were however rare.

b) Socio-demographic factors

Doctors’ personal backgrounds and socio-demographic factors (e.g. age, gender, marital status) have received considerable research attention. Research indicates that younger doctors are more likely and willing to leave posts, with the reasons commonly cited being openness to change, actively seeking better job and/or training opportunities, and a desire to travel, alongside having a strong interest in practising medicine abroad (Heponiemi *et al.*, 2008; Ali Jadoo *et al.*, 2015; Lu *et al.*, 2017; Oh and Kim, 2019). Conversely, older doctors have been shown as less likely to leave because they experience greater work stability, enhanced promotional opportunities, and higher income levels, along with stronger personal ties and job satisfaction. Some older doctors were also reluctant to leave either because they were nearing their retirement period, or because of the potential negative impact of turnover on their retirement plans (Lachman and Noy, 1997; Wang *et al.*, 2015; Pantenburg *et al.*, 2018).

Older age did, however, appear to be associated with increased turnover within fast-paced, highly specialised clinical and acute departments (e.g. surgery and anaesthetic), and a higher proportion of doctors from these specialities were likely to look to leave as they got older (Wang *et al.*, 2015). It was suggested that this may potentially be in response to deteriorating relative performance owing to the value of doctors’ surgery skillset being diminished (Sherwood and Bismark, 2020).

Male doctors appeared more willing to leave (Ali Jadoo *et al.*, 2015; Brugha *et al.*, 2021), while married women with children were found to be less likely to leave (Pantenburg *et al.*, 2018). Researchers have attributed these findings to the fact that women are likely to have more social obligations and are therefore less geographically flexible and mobile than their male counterparts (Pantenburg *et al.*, 2018). Female doctors were also more inclined than their male counterparts to leave due to work-family conflicts generated by the suitability of work to their family demands. One such factor is the impact of overtime, which has been shown to be an important turnover predictor amongst female doctors due to the restrictions it can put on time available to spend with family (Ochsmann, 2012; Wu *et al.*, 2018). Marriage appeared to have a wider impact, in that married doctors (both men and women) were found to have less intention to leave. This may be due to a dual career effect. Similar findings were found amongst those with children, i.e. hospital doctors with kids were less likely to leave roles, particularly if they were currently living close to family members such as grandparents which was a heavily cited factor of influence (Wu *et al.*, 2018; Brugha *et al.*, 2021).

### Meso level factors

There were 43 studies that examined how organisational factors impact voluntary hospital doctor turnover. We identified ten sub-themes within this data set with the most attention (22 studies) given to the role of the overall organisational environment and culture. These studies varied in terms of the gravity of the situation faced by doctors. For example, some studies highlighted unsafe working environments that featured work violence (Ali Jadoo *et al.*, 2015; Lu *et al.*, 2017), high levels of workplace bullying (Walsh, 2013; Clarke *et al.*, 2017; Lambert *et al.*, 2018), and frequent incidences of medical disputes where doctors were predominantly unsatisfied with the response of the hospital system. This led doctors feeling uncomfortable, threatened, and insecure (Wang *et al.*, 2015; Zhang *et al.*, 2019).

Poor working relationships, a lack of teamwork, low levels of worker engagement, and significant communication barriers with other hospital staff members (e.g. other doctors, nurses, and lab assistants) also emerged as factors contributing to doctor turnover within hospitals. These aspects were seen to create a poor atmosphere at work characterised by limited interactions and reduced or absent harmony within the unit/ward/hospital (Walsh, 2013; Wang *et al.*, 2015; Clarke *et al.*, 2017; James and Gerrard, 2017; Pantenburg *et al.*, 2018; Smith *et al.*, 2018, p. 2; Wu *et al.*, 2018; Duan *et al.*, 2019; Martinussen *et al.*, 2020). Conversely, a positive working environment characterised by high morale, flexibility, and non-hierarchical team structures emerged as positive for doctors’ intention to stay and work towards lessening workplace conflict (Lachman and Noy, 1997)

The next most considered factor was the quality of management, supervisory support, and leadership style within the workplace; 19 studies emphasised the effects of lack of support from management which can lead to doctors not feeling listened to, valued, or recognised (e.g. James and Gerrard, 2017; Roy *et al.*, 2017; Zhang *et al.*, 2017; Lambert *et al.*, 2018; Duan *et al.*, 2019; Martinussen *et al.*, 2020). Situations, where performance evaluations were lacking emerged as important (e.g. Ochsmann, 2012; Clarke *et al.*, 2017; Lu *et al.*, 2017; Smith *et al.*, 2018; Brugha *et al.*, 2021; Byrne *et al.*, 2021) in terms of identifying a lack of supervisory support and/or mentoring from consultants or senior doctors. This negatively impacted doctor turnover. Doctors highlighted issues around limited informational support (work-related information, appraisals, feedback), material support (assistance, time, guidance) and emotional support (care, acceptance, helping) from managers and supervisors that led to withdrawal from work. Conversely, high levels of supervision, support, and recognition reduced turnover (Brugha *et al.*, 2021). In one study, the probability that doctors would show an intention to leave was one and a half times lower where leaders were characterised as having a professionally supportive leadership style (Martinussen *et al.*, 2020). The research indicates that poor leadership increases turnover (Zhang and Feng, 2011; Martinussen *et al.*, 2020).

In 28 studies, educational and career advancement opportunities were shown to influence turnover (e.g. Ochsmann, 2012; Degen *et al.*, 2014; Clarke *et al.*, 2017; Zhang *et al.*, 2017; Lambert *et al.*, 2018; Pantenburg *et al.*, 2018; Duan *et al.*, 2019; Pflipsen *et al.*, 2019). These studies demonstrated how poor training programmes and pathways, including limited postgraduate training opportunities (mandatory to becoming a consultant) were especially problematic. Inadequate formal training accompanied by a lack of informal teaching, supervision, guidance, mentoring, coaching, and career advice from the supervisory team were all reported as causing doctors to look at options elsewhere (Smith *et al.*, 2018; Duan *et al.*, 2019). Informal learning was also noted to have a positive impact on patient safety, and so the potential effects of its absence go beyond the realm of doctor turnover.

Working hours were found to raise significant concerns for doctors around their health and well-being. Hospital doctors were found to struggle in managing the hours they were expected to undertake, especially when other job challenges were factored in; high job demands included clinical demands, patient load, care, decision-making about patients, academic responsibilities (examinations, research, acquiring new knowledge, and learning technologies), and administrative demands (paperwork, processing patients’ records) were regularly cited issues. When these are coupled with limited resources (Heponiemi *et al.*, 2016; James and Gerrard, 2017; Lambert *et al.*, 2018; Duan *et al.*, 2019; Brugha *et al.*, 2021; Byrne *et al.*, 2021), staffing issues (Roy *et al.*, 2017; Humphries *et al.*, 2018), and inflexible rota allocations (Fuß *et al.*, 2008; Wang *et al.*, 2015; Valle *et al.*, 2016), there was an inevitable (negative) impact on turnover. Research further indicates that long working hours appear as more detrimental amongst acute speciality doctors, such as surgical, emergency medicine, and intensive care doctors (Wang *et al.*, 2015; Tsai *et al.*, 2016; Wu *et al.*, 2018).

Several studies focussed on doctors’ pay and rewards (Zhang and Feng, 2011; Tsai *et al.*, 2016; Lu *et al.*, 2017; Zhang *et al.*, 2017; Lambert *et al.*, 2018; Pantenburg *et al.*, 2018; Wu *et al.*, 2018; Oh and Kim, 2019; Pflipsen *et al.*, 2019) and while these factors were relevant, they did not tend to be a major determinant of turnover (Zhang *et al.*, 2017). Such matters have not generally been shown to effectively moderate the adverse effects of long working hours on voluntary turnover (Tsai *et al.*, 2016). It was reported that pay was at times wholly insufficient to compensate for the workload, long hours, and skills used (Oh and Kim, 2019).

### Methodological approaches

The dominant methodology employed in this research area has been quantitative research (*n* = 44). A mere five papers that emerged through our systematic search were qualitative studies (see James and Gerrard, 2017; Humphries *et al.*, 2018; Smith *et al.*, 2018; Humphries *et al.*, 2019; Byrne *et al.*, 2021), with a mere two studies designed using mixed-method approach (Clarke *et al.*, 2017, Luboga *et al.*, 2011).

Within the quantitative studies, cross-sectional, self-administered surveys were the most common research tools (*n* = 25). Response rates varied from 14.3% to 95.5%, with the average across studies being 54.9%. Notably, 12 studies failed to report the response rate which raises concerns about the quality, relevance, and generalisability of the findings.

Turning to the qualitative studies, all five papers relied on semi-structured, in-depth interviews. While numbers are much less relevant in qualitative research, the number of interviews in each study ranged from 10 to 51.

Almost half of all studies (47%) drew on data from European countries, with Ireland being the country with the largest single number of published papers (*n* = 7), followed by the UK (*n* = 6). The next most prominent region for empirical work was Asia (*n* = 11), with China dominating in terms of country context (*n* = 6). There were seven studies from the US, four from the Middle East, and three each from Australia and Africa.

## Discussion and future research agenda

Our systematic review highlighted many contributing factors towards why hospital doctors voluntarily leave their jobs. As such, there is a relatively extensive body of research on hospital doctor turnover and in particular on the push factors leading to doctors leaving their job. However, extant literature, is very much dominated by cross-sectional, quantitative studies which raises concerns over the real explanatory power of existing research, especially in trying to make causal links. In addition, the lack of in-depth studies and which provide the lived experiences are notably lacking.

We identify several avenues for advancing knowledge on the turnover decisions of doctors. We know that hospital doctors are keen on some of the key components of their roles and are strongly committed to their profession due to the intrinsic value they derive from what they do (e.g. patient care). These factors remain important, despite much dissatisfaction with meso-level problems such as working hours, shift work patterns, and administrative aspects of their jobs. While empirical research emanates from many different country contexts, the research field lacks comparative studies, which could foster a wider understanding of how macro and meso factors interact. This area of interest may arguably become more relevant in the ongoing aftermath of the Covid-19 pandemic. Some pertinent questions include, what, if any, impact has the pandemic had on the commitment and satisfaction of hospital doctors? What, if any, impact did different public policy responses have on patients, hospital operations, working conditions, and individual workers’ intentions to stay in or leave their jobs?

A further area that we see as a future research domain is the idea of collective doctor turnover. The extant employee turnover literature has, unsurprisingly, been heavily focussed on the individual level of analysis (Hausknecht, 2017; Hom *et al.*, 2017). There has been less consideration of collective turnover, which may be particularly important within some national contexts and in relation to junior doctors. It may be important to assess to what extent is there a contagion effect (Porter and Rigby, 2021) amongst junior doctors in hospital contexts where there is much frustration over working conditions. In the Irish hospital context, for example, it would be useful to consider the impact on retention from issues junior doctors face around wages and overtime payments linked to compulsory six-month rotations (McGowan *et al.*, 2013). It may also be useful to assess if wider collective discord amongst a group of doctors has knock-on effects on turnover amongst other healthcare professionals.

We also note how much less consideration was found regarding understanding why doctors stay in their jobs and within their organisations. The question of why employees stay or continue to remain in their jobs has been gaining much attention and traction in the wider employee turnover literature (Mitchell *et al.*, 2001; Holtom *et al.*, 2008) but this appears largely absent with respect to hospital doctors (notable exception being Valle *et al.*, 2016). This paper illustrated that an individual’s links to others in the workplace, their fit with their job, and the sacrifice that would result from quitting their role impacted on stay decisions. More specifically, this study found that, although doctors were not satisfied with their job’s extra shifts (nights, weekends, and holidays) and the non-clinical aspects of their work (administrative aspects), they were looking to stay in their roles. This finding was ascribed to doctors’ high level of embeddedness in their roles which led to them planning on staying rather than looking to leave. We argue that there is much merit in researchers focussing on why doctors stay because it can offer a more holistic understanding of what can be done by policymakers and practitioners charged with retaining hospital doctors.

The retention dimension in the extant employee turnover literature draws heavily from the job embeddedness theory (Mitchell *et al.*, 2001), which seeks to explain why an employee stays in their job based on the level of fit, links, and relationships with the organisation and community. Job embeddedness includes many factors (on the job and off the job) which could play a role in an employee’s decision to stay. There is a need to consider, not just work-related factors, but also the non-work domain, given that off-the-job embeddedness may play a key role. When one factors in the international diversity of hospital doctors in many health systems this may be especially important. Research on hospital doctor turnover has largely tended to ignore the role of non-work factors. Non-work generally refers to activities and responsibilities within the family domain, as well as to obligations and events considered as interests and/or duties outside the workplace environment, in which employees routinely find themselves involved. These activities involve important commitments linked to household activities, caregiving responsibilities, and social obligations. Although some studies in our review (Lambert *et al.*, 2018; Pantenburg *et al.*, 2018) mentioned that hospital doctors seek improvements to their quality of life, what this means for retention prospects is unclear.

In terms of the methodological approaches and future research we call for an increased focus on in-depth, qualitative approaches. These approaches can bring a more nuanced understanding of the lived experiences of hospital doctors and their decision-making around staying or leaving. The exploration of meaning deriving from the words, experiences, and imagery shared by doctors can be crucial for the understanding of hospital workplaces (Cassell *et al.*, 2018). Qualitative approaches can therefore enable us to grasp the paradoxical tensions and challenges around relationships, power dynamics, and other macro, micro, and meso factors. A larger body of qualitative data might help us move towards a deeper understanding of the context of hospital doctors’ working lives (Eriksson and Kovalainen, 2008; Ruel, 2017) than the (dominant) cross-sectional quantitative designs.

Finally, we suggest that researchers should move beyond solely traditional semi- or unstructured interview approaches to experiment with novel approach such as diary studies. Diaries are a “method to collect data at the daily level or even several times a day” (Ohly *et al.*, 2010, pp. 79–93). Through this approach, researchers may be able to collect more real-time, work-related experiences from doctors and gain improved understanding of how static or dynamic their thoughts, feelings, and behaviours may be and how that builds up to influence retention and/or turnover decision-making (Kai Christian, 2017). The diary studies method offers more precise and frequent data collection in a natural context (Bakker and Xanthopoulou, 2009), thus enabling the researcher to capture “life as it is lived” (Bolger *et al.*, 2003, p. 597). This approach speaks to our final methodological call for a greater focus on longitudinal research designs. While cross-sectional designs offer economic advantages, long-term and multi-stage data collection will help advance our understanding of some complex questions, and better appreciate the influence and interplay of macro, micro and meso factors.

We also call on researchers to be more cognisant of the quality of their research design and the information they present to the reader. Papers should provide the key information that enables the reader to evaluate the legitimacy, depth, and breadth of the findings. Notable concerns arose in relation to many quantitative papers where there was, for example, a failure to provide details on response rates. In addition, researchers may wish to reflect on the utility of turnover intentions as a proxy metric considering recent concerns in this area (Purl *et al.*, 2016; Bolt *et al.*, 2022).

## Practice implications

Hospital doctor turnover is a challenge faced by many countries globally. The study of employee turnover within healthcare settings is well-developed (Bolt *et al.*, 2022), and this review indicates that many variables have been found to affect hospital doctor turnover. So, what does our review and future research considerations mean, both for practice, and for those who are charged with managing doctor retention? Firstly, we would argue that in-depth understanding of doctor turnover remains limited due to methodological limitations and the failure to consider retention to any great degree. While many human resource officers have heard of and undertake exit interviews, fewer consider the use of “stay interviews”. Although you can gather a lot of relevant information through performance reviews and informal discussions, a focussed and targeted conversation on retention may be worth considering amongst progressive HR professionals within hospital environments. This conversation, which might involve line managers (Budworth *et al.*, 2015), would centre around what motivates a doctor to stay, what could be done to better their work experience, how they envision their career developing, and what supports they desire. Stay interviews may shed light on the issues raised earlier about contagion and collective turnover, and they may also provide ways to gather input from close co-workers of a doctor who has already decided to exit, providing information to inform proactive retention measures.

Hospital management and recruiters should also consider off-the-job components when evaluating ways to support doctors. For example, what can they do to assist new doctors in making off-the-job connections? Often, the links that exist through relationships at work emerge organically and are left to the individuals themselves. However, these social connections do not always emerge easily, and therefore, there is scope for employers to actively connect people to one another.

## Figures and Tables

**Table 1:** List of journals published

Journal	No. of papers
*BMC Health Services Research*	8
*Human Resources for Health*	4
*BMJ Open*	3
*Health Policy*	3
*Health Care Management Review*	2
*Journal of the Royal Society of Medicine*	2
*International Journal of Health Policy Management, International Journal of Health Planning and Management, BMC Medical Education, BMC Medicine, New Zealand Journal of Human Resource Management, Academy of Health Care Management Journal, International Health Hospital and Health service Administration, International Journal of Environment Research and Public Health, Gender, Work and Organization, Journal of Family Practice, Journal of work and organizational Psychology, Journal of Business Inquiry, Psicothema, Academic Medicine, Emergency Medicine International, Health and Quality Life Outcomes, Journal of the American College of Surgeons, Irish Journal of Medical Science, BMC Public Health, The Irish Medical Journal, The New Zealand Medical Journal, Australian Health Review, Journal of the Formosan Medical Association, Journal of the American College of Surgeons, Scandinavian journal of work, environment* & *health, Emergency Medicine Journal, Psychology, Health and Medicine, International Journal of Public Administration*	All 1
Total Papers	51

**Source(s):** Authors’ original research data/created by the authors

**Table A1:** Overview of articles

Author(s) and year	Research aims	Keywords	Methodology	Key findings
Lambert *et al.* (2018)	To report the reasons why doctors are considering leaving medicine or the UK	Physicians, career choice, medical staff, attitude of health personnel, emigration, travel	Quantitative	Increasing negative views are held by many doctors about many aspects of the experience working as a junior doctor in the NHS and the difficulty of delivering high-quality care within the under-funded system
Ali Jadoo *et al.* (2015)	To explore prominent factors affecting turnover amongst Iraqi doctors	Iraqi doctors, health human resources migration, turnover intention, job satisfaction	Quantitative	The high turnover intention amongst Iraqi doctors is significantly associated with poor working and security conditions
Humphries *et al.* (2019)	To assess how deteriorating job quality and the normalisation of extreme working is driving Irish trained hospital doctors to leave	Medicine, migration, job quality, austerity qualitative, extreme work	Qualitative	The deterioration in medical job quality and the normalisation of extreme working is a key driver of doctor emigration from Ireland and deters return
Zhang and Feng (2011)	To analyse the relationship between job satisfaction, burnout, and turnover intention, and to determine burnout as a mediator amongst Chinese physicians from urban state-owned medical institutions	Occupational burnout, job satisfaction, turnover intention, Chinese physicians	Quantitative	There may be partial mediation effects of occupational burnout, mainly through emotional exhaustion, within the impact of job satisfaction on turnover intention
Pantenburg *et al.* (2018)	To provide current data in an effort to promote the identification of 'push' factors motivating German physicians to emigrate	Physician migration, ‘push’ factors, job satisfaction, physician attrition	Quantitative	Government subsidy per bed, personnel funding per capita, the number of physicians per bed and the number of hospital medical disputes significantly affected physicians’ intention to leave
Oh and Kim (2019)	To investigate the turnover intentions of employed doctors in Korea to provide evidence for policies to prevent and control their turnover intentions	Employed doctors, turnover intentions, Korean Physician Survey	Quantitative	Turnover intentions because of low-quality work conditions and poor environments to maintain their position, thus deteriorating their job security
Moss *et al.* (2004)	To study the reasons given by junior doctors trained in the United Kingdom for considering leaving UK medicine	The UK	Quantitative	Junior doctors wish to work abroad, but to stay in medicine. This is due to preference for different lifestyles, outside of the United Kingdom
Walsh (2013)	To identify how issues relating to the work–life interface affect the wellbeing of hospital doctors and, in particular, their levels of job burnout and intentions to quit	The UK	Quantitative	Female doctors were more likely to experience job burnout than male doctors. Aspects of work-life interface affect the wellbeing of all doctors but women tend to rely on different forms of social support than men
Pathman *et al*. (2002)	To better understand the relationship between physicians’ satisfaction with various aspects of their work and whether or not they have thoughts or plans to leave their jobs	The USA	Quantitative	The aspects of work for which dissatisfaction is associated with plans for leaving differ somewhat for generalists and specialists, and for physicians at various stages of their careers; and anticipated job departure is more common amongst physicians who are relatively dissatisfied with any of a variety of aspects of their work
Williams *et al*. (2010)	To provide empirically based evidence upon which recommendations can be made to physicians, managers, and policymakers regarding physicians’ intentions to withdraw from practice	The USA	Quantitative	Higher perceived stress is associated with lower satisfaction levels that are related to greater intentions to quit. Physicians experiencing burnout, anxiety, and depression seem to deal with these problems by leaving patient care in some way
Roy *et al*. (2017)	To investigate the effects of work characteristics and other predictors on job satisfaction, turnover intention, and burnout in doctors	Bangladesh	Quantitative	Organisational support was the strongest predictor adversely affecting job satisfaction, turnover intention and burnout of both public and private doctors; private doctors experienced more support
Degen *et al*. (2014)	To identify to what extent junior doctors’ training and working conditions determine their intention to leave clinical practice	Germany	Quantitative	Junior doctors undergoing speciality training experience high workload in hospital practice, which in turn influences their intentions to leave
Tsai *et al*. (2016)	To analyse the effect of work hours on turnover intention, and also puts pay satisfaction into consideration to estimate its possible moderating effect	Taiwan	Quantitative	Work hours exhibited an independent relationship with turnover intention. Pay satisfaction could not effectively moderate the positive relationship between work hours and intentions to leave a doctor’s current hospital
Martinussen *et al*. (2020)	To investigate hospital physicians’ intention to leave their current job and whether such intentions are associated with how physicians assess their leaders and the organisational context	Norway	Quantitative	A professional, supportive leadership style has a positive influence on the retention of physicians in public hospitals
Sharma *et al*. (2012)	To investigate factors which influenced UK-trained doctors to emigrate to New Zealand and factors which might encourage them to return	The UK	Quantitative	Reasons for emigration; job satisfaction; satisfaction with leisure time; intentions to stay in New Zealand; and changes to the UK NHS which might increase the likelihood of return
Lu *et al*. (2017)	To investigate the relationship between job satisfaction, work stress, work–family conflict and turnover intention, and explores factors associated with turnover intention amongst physicians in Guangdong Province, China	China	Quantitative	Job satisfaction, work stress, work-–family conflict, hours worked per week, working in an urban/rural area, types of institution, and age are influencing factors on turnover intention
Tziner *et al*. (2015)	To examine the relationship between perceived work stress, burnout, satisfaction at work, and turnover intention	Israel	Quantitative	Physicians are required to cope with numerous demands: clinical, administrative, and academic – which easily results in various pressures, burnout, and intention to leave
Valle *et al*. (2016)	To test the job embeddedness construct with a sample of 183 Pediatric Emergency Medicine (PEM) physicians	The USA	Quantitative	Job embeddedness – a composite variable measuring physicians’ links to other people/the organisation, job fit, and the sacrifices inherent in job change – is inversely related to the turnover intentions of PEM physicians
Smith *et al*. (2018)	To explore the reasons that doctors choose to leave UK medicine after their foundation year two posts	Scotland	Qualitative	Availability of jobs elsewhere; a desire to improve work-life balance; or a desire to enjoy better job perks are the main reasons doctors leave
Moreno-Jiménez *et al.* (2012)	To examine how the moderating effect of commitment depends on difficult doctor–patient relations	Spain	Quantitative	General assumption that commitment has a unilateral negative effect, and difficult patients have a positive effect on turnover intentions
Ochsmann (2012)	To examine the association between workplace factors and thinking about leaving clinical care by junior doctors in Germany, and used a gender-stratified approach to address the so-called feminisation of medicine	Germany	Quantitative	Workplace factors predict the wish to leave clinical care for junior doctors. Male and female junior doctors seem to have different priorities in the workplace, which should be addressed in order to retain them in patient care
Wu *et al*. (2018)	To investigate the relationships amongst intention to leave, emergency physician clinical activities, work–family conflicts, and gender differences in emergency physicians	Taiwan	Quantitative	Females and EPs with higher levels on the WIF scale (Work interfering with family) were more likely to leave emergency clinical practice
Gauld and Horsburgh (2015)	To probe the motives of UK-trained doctors who have migrated to New Zealand, their experiences in New Zealand and reasons for departing again	New Zealand	Quantitative	Motivated by ‘pull’ factors that also motivate IMGs moving from lower to higher income countries: quality of life, better working conditions and career opportunities
Humphries *et al*. (2018)	To explore the generational component of Ireland’s failure to retain doctors	Ireland	Qualitative	The new generation of doctors differs from previous generations in several distinct ways: poor training and practice, and an under- staffed health system
Pflipsen *et al*. (2019)	To gain insights into the reasons for attrition from EM training in Ireland	Ireland	Quantitative	The need to improve training and working conditions in Emergency Medicine in Ireland to reduce attrition and improve retention of EM staff
Brugha *et al*. (2021)	To measure junior doctors’ migration intentions, the reasons they leave, and the likelihood of them returning	Ireland	Quantitative	Ireland’s doctor retention strategy has not addressed the root causes of poor training and working experiences in Irish hospitals
Clarke *et al*. (2017)	To measure and explore the predictors of trainee doctor emigration from Ireland	Ireland	Mixed method	Large-scale dissatisfaction with working conditions, training, and career opportunities influences doctors migration
Fuß *et al.* (2008)	To investigate predictors for one particular direction of Work-Family Conflict – namely work interfering with family conflict (WIF) – which are located within the psychosocial work environment or work organisation of hospital physicians	Germany	Quantitative	Work interfering with family conflict (WIF) as part of work-family conflict (WFC) was highly prevalent amongst German hospital physicians. Factors of work organisation as well as factors of interpersonal relations at work were identified as significant predictors for WIF.
Bruce-Brand *et al*. (2012)	To establish levels of satisfaction, sources of dissatisfaction, and the major reasons for junior doctors seeking work abroad	Ireland	Quantitative	Multiple sources of dissatisfaction: the state of the health care system, staffing cover for leave and illness, the dearth of consultant posts, the need to move around Ireland, the long hours worked and the degree of work-related stress
Morton and Karen Schaab (2008)	To provide information about the number of non-consultant hospital doctors leaving a hospital’s employ over a 12-month period; the reasons for their departure	New Zealand	Quantitative	Attracted to their next workplace, related to a wish to work somewhere new, rather than representing any dissatisfaction with their previous organisation or working conditions
Zhang *et al.* (2017)	To explore prominent factors affecting turnover intentions amongst public hospital doctors in urban areas, particularly in Xiangyang City, Hubei Province, a middle-level city in central China	China	Quantitative	Dissatisfaction with working conditions and hospital management processes, as well as work pressures, were significant factors contributing to the turnover intentions of public hospital doctors
Wang *et al*. (2015)	To assess the working conditions of anaesthesiologists in Taiwan and their satisfaction with their occupation, and to identify the factors associated with the intentions to leave	Taiwan	Quantitative	Unfavourable working conditions were considered to lower the satisfaction of anaesthesiologists in Taiwan. In particular, an inability to take care of the family and a low salary were major factors in deterring anaesthesiologists in Taiwan from continuing in anaesthesia
Duan *et al*. (2019)	To identify the prevalence of workplace violence; to examine the association between exposure to WPV, job satisfaction, job burnout and turnover intention of Chinese physicians; and to verify the mediating role of social support	China	Quantitative	The results show a high prevalence of workplace violence in Chinese tertiary hospitals. Social support was a partial mediator between WPV and job satisfaction, as well as burnout and turnover intention
Opoku and Apenteng (2014)	To identify the relationship between career satisfaction and the intention of active Ghanaian physicians to leave the country within the next 5 years	Ghana	Quantitative	Physicians who were house officers or medical officers and those who reported dissatisfaction with their compensation were more likely to report that they were thinking about leaving Ghana within the next 5 years
Mousavi *et al.* (2019)	To identify job satisfaction and turnover intention amongst anaesthesiologists in Iran	Iran	Quantitative	Significant association was found between job satisfaction and anaesthesiologists’ intention to leave their current employment
Heponiemi *et al*. (2014)	To examine the prospective associations of work-related physical violence and bullying with physicians’ turnover intentions and job satisfaction	Finland	Quantitative	Violence led to increased physician turnover intentions and both bullying and physical violence led to reduced physician job satisfaction, even after adjustments. Opportunities for job control were able to alleviate the increase in turnover intentions resulting from bullying
Mathieu and Mathieu (2011)	To measure the role of WFC in the intention to leave the job in medical residents	Canada	Quantitative	Work–family conflict explains 22% of the variance in the intention of the medical residents to quit their job
Mahoney *et al.* (2020)	To evaluate the state of our surgical workforce by exploring current practice patterns, job satisfaction, and reasons why surgeons consider leaving surgery	The USA	Quantitative	Work time requirements and lack of personal time are leading factors contributing to surgeons leaving practice, though their satisfaction towards being a surgeon is high
Luboga *et al.* (2011)	To identify what could entice physicians to stay longer and improve satisfaction with current positions and future career intentions	Africa	Mixed method	Sources of dissatisfaction amongst physicians were quality of management, availability of equipment and supplies, quality of facility infrastructure, staffing and workload, political influence, community location, and professional development
Lachman and Noy (1997)	Examines the effects of physicians standing within their hospital membership	Israel	Quantitative	Factors reflecting the physicians’ standing within the hospital were the main predictors of this anticipation that doctors would retain their hospital membership in the long term
Haar and Edward (2013)	Examine the factors driving hospital doctors from their profession	New Zealand	Quantitative	Perceived organisational support was positively related to job satisfaction, which in turn was negatively related to emotional exhaustion, cynicism, stress, and profession turnover
Heponiemi *et al*. (2008)	To examine whether active on-call hours and the co-occurrence of lifestyle risk factors are associated with physicians' turnover intentions and distress	Finland	Quantitative	On-call duty and the occurrence of lifestyle risk factors may both decrease physicians’ well-being and increase their intentions to leave their jobs
Masselink *et al.* (2008)	To examine physicians’ positive relationships with colleagues, staff, and patients and their relationship to withdrawal from practice. Do the effects of these relational factors differ for large-group and solo/small-group practice physicians?	The USA	Quantitative	Relationships with colleagues had a significant and negative association with intended withdrawal from practice for large-group practice physicians. The relationships with colleagues, staff, and patients was significant for large-group practice physicians, but they only approached significance for solo/small-group practice physicians
Domagała and Dubas-Jakóbczyk (2019)	To evaluate the scale of migration intentions amongst physicians practicing in Polish hospitals	Poland	Quantitative	Higher earnings, better working conditions, and better work-life balance abroad were correlated with intention to leave. Age, and higher career satisfaction were negatively related to the intention to migrate
James and Gerrard (2017)	To explore the views of current EM consultants on positive and negative aspects of their work to help prospective trainees	The UK	Qualitative	The high-pressured EM environment was a key motivator for an EM career; however, there is concern over the sustainability of this long term, with a risk of career burnout due to lack of transition into ‘wind-down’ career pathways leading to them deciding to stay or leave
Heponiemi *et al*. (2016)	Investigated whether having on-call duties is associated with physicians’ turnover intention and whether job strain variables moderate this association	Finland	Quantitative	The results showed that job strain moderated the association between being on-call and turnover intention. The highest levels of turnover intentions were amongst those who had a high level of on-call duties
Ma *et al.* (2019)	To examine the moderator effect of pragmatism on the relationship between commitment HRM policies and turnover intention via DPR.	China	Quantitative	Commitment to HR practices is positively associated with the DPR and, overall, DPR is negatively related to turnover intention. Pragmatism moderates the association between DPR and turnover intention
Byrne *et al*. (2021)	To understand how the organisation of medical work shapes the everyday work experiences underpinning doctor migration trends in the case of Irish-trained emigrant doctors in Australia	Ireland	Qualitative	Retention of hospital doctors is as much about the quality of the work experience as it is about the quantity and composition of the workforce
Hamidi *et al*. (2018)	To examine the associations between physician self-reported burnout, intent to leave (ITL) and actual turnover within two years, and 2) to estimate the cost of physician turnover attributable to burnout	The USA	Quantitative	Physicians who are experiencing burnout are more than twice as likely to leave their practice, and the effect of burnout on turnover is independent of personal factors such as anxiety or depression
Gutacker *et al*. (2019)	To investigate the effect of medical revalidation on the rate at which consultants leave NHS practice and assess any differences between the performance of consultants who left or remained in practice before and after the introduction of revalidation	The UK	Quantitative	Revalidation has led to high numbers of doctors ceasing clinical practice, over and above other contemporaneous influences. Those ceasing clinical practice do not appear to have provided lower quality care, as approximated by mortality rates, when compared with those remaining in practice

**Source(s):** Authors’ original research data/created by the authors

## Appendix

Table A1

## References

[ref001] Ali Jadoo, S.A., Aljunid, S.M., Dastan, I., Tawfeeq, R.S., Mustafa, M.A., Ganasegeran, K. and AlDubai, S.A.R. (2015), “Job satisfaction and turnover intention amongst Iraqi doctors – a descriptive cross-sectional multicentre study”, Human Resources for Health, Vol. 13 No. 1, p. 21, doi: 10.1186/s12960-015-0014-6. 25903757 PMC4407309

[ref002] Bakker, A.B. and Xanthopoulou, D. (2009), “The crossover of daily work engagement: test of an actor-partner interdependence model”, Journal of Applied Psychology, Vol. 94 No. 6, pp. 1562-1571, doi: 10.1037/a0017525. 19916663

[ref003] Banerjee, D.S. and Gaston, N. (2004), “Labour market signalling and job turnover revisited”, Labour Economics, Vol. 11 No. 5, pp. 599-622, doi: 10.1016/j.labeco.2003.10.001.

[ref004] Bolger, N., Davis, A. and Rafaeli, E. (2003), “Diary methods: capturing life as it is lived”, Annual Review of Psychology, Vol. 54 No. 1, pp. 579-616, doi: 10.1146/annurev.psych.54.101601.145030. 12499517

[ref005] Bolibar, M. (2016), “Macro, meso, micro: broadening the ‘social’ of social network analysis with a mixed methods approach”, Quality and Quantity, Vol. 50 No. 5, pp. 2217-2236, doi: 10.1007/s11135-015-0259-0.

[ref006] Bolt, E.E.T., Winterton, J. and Cafferkey, K. (2022), “A century of labour turnover research: a systematic literature review”, International Journal of Management Reviews, Vol. 24 No. 4, pp. 555-576, doi: 10.1111/ijmr.12294.

[ref085] Bramer, W., De Jonge, G., Rethlefsen, M., Frans, M. and Jos, K. (2018), “A systematic approach to searching: an efficient and complete method to develop literature searches”, Journal of the Medical Library Association, Vol. 106 No. 4, pp. 531-541, doi: 10.5195/jmla.2018.283. 30271302 PMC6148622

[ref007] Bruce-Brand, R., Broderick, J., Ong, J. and O’Byrne, J. (2012), “Diagnosing the doctors’ departure: survey on sources of dissatisfaction amongst Irish junior doctors”, Irish Medical Journal, Vol. 105 No. 1, pp. 15-18. 22397207

[ref008] Brugha, R., Clarke, N., Hendrick, L. and Sweeney, J. (2021), “Doctor retention: a cross-sectional study of how Ireland has been losing the battle”, International Journal of Health Policy and Management, Vol. 10 No. 6, pp. 299-309, doi: 10.34172/ijhpm.2020.54. 32610753 PMC9056149

[ref009] Budworth, M.-H., Latham, G.P. and Manroop, L. (2015), “Looking forward to performance improvement: a field test of the feedforward interview for performance management”, Human Resource Management, Vol. 54 No. 1, pp. 45-54, doi: 10.1002/hrm.21618.

[ref010] Byrne, J.-P., Conway, E., McDermott, A., Matthews, A., Prihodova, L., Costello, R. and Humphries, N. (2021), “How the organisation of medical work shapes the everyday work experiences underpinning doctor migration trends: the case of Irish-trained emigrant doctors in Australia”, Health Policy, Vol. 125 No. 4, pp. 467-473, doi: 10.1016/j.healthpol.2021.01.002. 33551205

[ref011] Cassell, C., Cunliffe, A.L. and Grandy, G. (2018), The SAGE Handbook of Qualitative Business and Management Research Methods: History and Traditions, SAGE Publications, Thousand Oak, California.

[ref012] Chojnicki, X. and Moullan, Y. (2018), “Is there a ‘pig cycle’ in the labour supply of doctors? How training and immigration policies respond to physician shortages”, Social Science and Medicine, Vol. 200, pp. 227-237, (1982), doi: 10.1016/j.socscimed.2018.01.038. 29425902

[ref013] Clarke, V. and Braun, V. (2017), “Thematic analysis”, The Journal of Positive Psychology, Vol. 12 No. 3, pp. 297-298, doi: 10.1080/17439760.2016.1262613.

[ref014] Clarke, N., Crowne, S., Humphries, N., Conroy, R.M., O'Hare, S. and Kavanagh, P. (2017), “Factors influencing trainee doctor emigration in a high income country: a mixed methods study”, Human Resources for Health, Vol. 15, pp. 1-12. 28942731 10.1186/s12960-017-0239-7 PMC5611654

[ref015] de Vries, N., Boone, A., Godderis, L., Bouman, J., Szemik, S., Matranga, D. and de Winter, P. (2023), “The race to retain healthcare workers: a systematic review on factors that impact retention of nurses and physicians in hospitals”, INQUIRY: The Journal of Health Care Organization, Provision, and Financing, Vol. 60, doi: 10.1177/00469580231159318. PMC10014988 36912131

[ref016] Degen, C., Li, J., Angerer, P. and Siegrist, J. (2014), “The impact of training and working conditions on junior doctors' intention to leave clinical practice”, BMC Medical Education, Vol. 14 No. 1, p. 119, doi: 10.1186/1472-6920-14-119. 24942360 PMC4068906

[ref097] Domagała, A. and Dubas-Jakóbczyk 2, K. (2019), “Migration intentions among physicians working in Polish hospitals - insights from survey research”, Health Policy, Vol. 123 No. 8, pp. 782-789, doi: 10.1016/j.healthpol.2019.06.008. 31279589

[ref017] Duan, X., Ni, X., Shi, L., ZhangYe, L.Y., Mu, H., Li, Z., Liu, X., Fan, L. and Wang, Y. (2019), “The impact of workplace violence on job satisfaction, job burnout, and turnover intention: the mediating role of social support”, Health and Quality of Life Outcomes, Vol. 17 No. 1, p. 93, doi: 10.1186/s12955-019-1164-3. 31146735 PMC6543560

[ref018] Eriksson, P. and Kovalainen, A. (2008), Qualitative Methods in Business Research, SAGE Publications, Thousand Oak, California.

[ref019] Fibuch, E. and Ahmed, A. (2015), “Physician turnover: a costly problem”, Physician Leadership Journal, Vol. 2 No. 3, pp. 22-25. 26214946

[ref020] Fuß, I., Nübling, M., Hasselhorn, H.-M., Schwappach, D.L.B. and Rieger, M.A. (2008), “Working conditions and work-family conflict in German hospital physicians: psychosocial and organisational predictors and consequences”, BMC Public Health, Vol. 8 No. 1, pp. 353-369, doi: 10.1186/1471-2458-8-353. 18840296 PMC2577658

[ref089] Gauld, R. and Horsburgh, S. (2015), “What motivates doctors to leave the UK NHS for a ‘life in the sun’ in New Zealand; and, once there, why don’t they stay?”, Human Resource for Health, Vol. 13 No. 7, doi: 10.1186/s12960-015-0069-4. PMC4563843 26350706

[ref021] Godlee, F. (2008), “Understanding the role of the doctor”, BMJ, Vol. 337 No. 7684, pp. 1425-1426, doi: 10.1136/bmj.a3035. 19097980

[ref022] Gutacker, N., Bloor, K., Bojke, C., Novielli, N. and Smith, P. (2019), “Does regulation increase the rate at which doctors leave practice? Analysis of routine hospital data in the English NHS following the introduction of medical revalidation”, BMC Medicine, Vol. 17 No. 1, p. 33, doi: 10.1186/s12916-019-1270-4. 30744639 PMC6371486

[ref095] Haar, J. and Edward, P. (2013), “Factors driving hospital doctors from their profession: evidence from New Zealand”, New Zealand Journal of Human Resources Management, Vol. 13 No. 2, p. 67.

[ref023] Hamidi, M.S., Bohman, B., Sandborg, C., Smith-Coggins, R., de Vries, P., Albert, M.S., Murphy, M.L., Welle, D. and Trockel, M.T. (2018), “Estimating institutional physician turnover attributable to self-reported burnout and associated financial burden: a case study”, BMC Health Services Research, Vol. 18 No. 1, p. 851, doi: 10.1186/s12913-018-3663-z. 30477483 PMC6258170

[ref024] Hausknecht, J.P. (2017), “Collective turnover”, Annual Review of Organizational Psychology and Organizational Behavior, Vol. 4 No. 1, pp. 527-544, doi: 10.1146/annurev-orgpsych-032516-113139.

[ref025] Heponiemi, T., Kouvonen, A., Vänskä, J., Halila, H., Sinervo, T., Kivimäki, M. and Elovainio, M. (2008), “Effects of active on-call hours on physicians' turnover intentions and well-being”, Scandinavian Journal of Work, Environment and Health, Vol. 34 No. 5, pp. 356-363, doi: 10.5271/sjweh.1278. 18853067

[ref026] Heponiemi, T., Kouvonen, A., Vänskä, J., Halila, H., Sinervo, T. and Kivimäki, M. (2014), “The prospective effects of workplace violence on physicians' job satisfaction and turnover intentions: the buffering effect of job control”, BMC Health Services Research, Vol. 14 No. 1, p. 19, doi: 10.1186/1472-6963-14-19. 24438449 PMC3898009

[ref027] Heponiemi, T., Presseau, J. and Elovainio, M. (2016), “On-call work and physicians' turnover intention: the moderating effect of job strain”, Psychology, Health and Medicine, Vol. 21 No. 1, pp. 74-80, doi: 10.1080/13548506.2015.1051061. 26072662

[ref028] Holtom, B.C., Mitchell, T.R., Lee, T.W. and Inderrieden, E.J. (2005), “Shocks as causes of turnover: what they are and how organizations can manage them”, Human Resource Management, Vol. 44 No. 3, pp. 337-352, doi: 10.1002/hrm.20074.

[ref029] Holtom, B.C., Mitchell, T.R., Lee, T.W. and Eberly, M.B. (2008), “Turnover and retention research: a glance at the past, a closer review of the present, and a venture into the future”, Academy of Management Annals, Vol. 2 No. 1, pp. 231-274, doi: 10.5465/19416520802211552.

[ref030] Hom, P.W., Lee, T.W., Shaw, J.D. and Hausknecht, J.P. (2017), “One hundred years of employee turnover theory and research”, Journal of Applied Psychology, Vol. 102 No. 3, pp. 530-545, doi: 10.1037/apl0000103. 28125259

[ref031] Humphries, N., Crowe, S. and Brugha, R. (2018), “Failing to retain a new generation of doctors: qualitative insights from a high-income country”, BMC Health Services Research, Vol. 18 No. 1, p. 144, doi: 10.1186/s12913-018-2927-y. 29486756 PMC5830046

[ref032] Humphries, N., McDermott, A.M., Conway, E., Byrne, J.P., Prihodova, L., Costello, R. and Matthews, A. (2019), “Everything was just getting worse and worse: deteriorating job quality as a driver of doctor emigration from Ireland”, Human Resources for Health, Vol. 17, pp. 1-11, doi: 10.1186/s12960-019-0424-y. 31815621 PMC6902557

[ref033] James, F. and Gerrard, F. (2017), “Emergency medicine: what keeps me, what might lose me? A narrative study of consultant views in Wales”, Emergency Medicine Journal, Vol. 34 No. 7, pp. 436-440, doi: 10.1136/emermed-2016-205833. 28356388

[ref034] Kai Christian, B. (2017), “Daily ethical leadership: insights from a diary study”, Academy of Management Proceedings.

[ref035] Keogh, B. (2013), Review into the Quality of Care and Treatment provided by 14 Hospital Trusts in England: Overview Report, National Health Service.

[ref036] Khan, A., Teoh, K.R., Islam, S. and Hassard, J. (2018), “Psychosocial work characteristics, burnout, psychological morbidity symptoms and early retirement intentions: a cross-sectional study of NHS consultants in the UK”, BMJ Open, Vol. 8 No. 7, e018720, doi: 10.1136/bmjopen-2017-018720. PMC6059335 30037857

[ref037] Kirch, D.G. and Petelle, K. (2017), “Addressing the physician shortage: the peril of ignoring demography”, JAMA: The Journal of the American Medical Association, Vol. 317 No. 19, pp. 1947-1948, doi: 10.1001/jama.2017.2714. 28319233

[ref038] Kulik, C.T., Treuren, G. and Bordia, P. (2012), “Shocks and final straws: using exit-interview data to examine the unfolding model's decision paths”, Human Resource Management, Vol. 51 No. 1, pp. 25-46, doi: 10.1002/hrm.20466.

[ref039] Lachman, R. and Noy, S. (1997), “Salaried physicians' intent to retain hospital membership: the effects of position and work attitudes”, Hospital and Health Services Administration, Vol. 42 No. 4, pp. 509-524. 10174463

[ref040] Lambert, T.W., Smith, F. and Goldacre, M.J. (2018), “Why doctors consider leaving UK medicine: qualitative analysis of comments from questionnaire surveys three years after graduation”, Journal of the Royal Society of Medicine, Vol. 111 No. 1, pp. 18-30, doi: 10.1177/0141076817738502. 29035667 PMC5784487

[ref041] Lee, T.W. and Maurer, S.D. (1997), “The retention of knowledge workers with the unfolding model of voluntary turnover”, Human Resource Management Review, Vol. 7 No. 3, pp. 247-275, doi: 10.1016/s1053-4822(97)90008-5.

[ref042] Lee, T.W. and Mitchell, T.R. (1991), “The unfolding effects of organizational commitment and anticipated job satisfaction on voluntary employee turnover”, Motivation and Emotion, Vol. 15 No. 1, pp. 99-121, doi: 10.1007/bf00991478.

[ref043] Lee, T.W. and Mitchell, T.R. (1994), “An alternative approach: the unfolding model of voluntary employee turnover”, The Academy of Management Review, Vol. 19 No. 1, pp. 51-89, doi: 10.5465/amr.1994.9410122008.

[ref044] Lichtenstein, R.L. (1984), “The job satisfaction and retention of physicians in organized settings: a literature review”, Medical Care Review, Vol. 41 No. 3, pp. 139-179, doi: 10.1177/107755878404100301. 10299831

[ref045] Lock, F.K. and Carrieri, D. (2022), “Factors affecting the UK junior doctor workforce retention crisis: an integrative review”, BMJ Open, Vol. 12 No. 3, e059397, doi: 10.1136/bmjopen-2021-059397. PMC8960457 35351732

[ref046] Lu, Y., Hu, X., Huang, X., Zhuang, Y., Wu, H., Chen, L., Hu, W., Zou, H. and Hao, Y.T. (2017), “The relationship between job satisfaction, work stress, work-family conflict, and turnover intention amongst physicians in Guangdong, China: a cross-sectional study”, BMJ Open, Vol. 7 No. 5, e014894, doi: 10.1136/bmjopen-2016-014894. PMC5566636 28501813

[ref086] Luboga, S., Hagopian, A., Ndiku, J., Bancroft, E. and McQuide, P. (2011), “Satisfaction, motivation, and intent to stay among Ugandan physicians: a survey from 18 national hospitals”, The International Journal of Health Planning and Management, Vol. 26 No. 1, pp. 2-17, doi: 10.1002/hpm.1036. 22392793

[ref098] Ma, S., Xu, X., Trigo, V. and Ramalho, N. (2019), “Managing doctor-patient relationships and turnover intention in Chinese hospitals with commitment HRM: the moderating role of pragmatism”, International Journal of Public Administration, Vol. 44 No. 1, pp. 1-10, doi: 10.1080/01900692.2019.1672725.

[ref094] Mahoney, S., Strassle, P., Schroen, A., Agans, R., Turner, P., Meyer, A., Freischlag, J. and Brownstein, M. (2020), “Survey of the US surgeon workforce: practice characteristics, job satisfaction, and reasons for leaving surgery”, Journal of the American College of Surgeons, Vol. 230 No. 3, pp. 283-293, doi: 10.1016/j.jamcollsurg.2019.12.003. 31931143

[ref047] Malone, P. (2020), Trends in Medical Workforce Supply and Retention, Policy Context, Ireland.

[ref048] March, J.G. and Simon, H.A. (1958), Organizations, Wiley.

[ref049] Martinussen, P.E., Magnussen, J., Vrangbæk, K. and Frich, J.A. (2020), “Should I stay or should I go? The role of leadership and organisational context for hospital physicians' intention to leave their current job”, BMC Health Services Research, Vol. 20 No. 1, pp. 1-9, doi: 10.1186/s12913-020-05285-4. PMC7212554 32393343

[ref096] Masselink, L., Lee, S.-Y. and Konrad, T. (2008), “Workplace relational factors and physicians’ intention to withdraw from practice”, Health Care Management Review, Vol. 33 No. 2, pp. 178-187, doi: 10.1097/01.HMR.0000304507.50674.28. 18360168

[ref093] Mathieu, C. and Mathieu, C. (2011), “The impact of work-family conflict on the intention to quit the job in a sample of medical residents”, Academy of Health Care Management Proceedings, Vol. 8 No. 2.

[ref050] McGowan, Y., Humphries, N., Burke, H., Conry, M.C., Morgan, K. and McAuliffe, E. (2013), “Through doctors' eyes: a qualitative study of hospital doctor perspectives on their working conditions”, British Journal of Health Psychology, Vol. 18 No. 4, pp. 874-891, doi: 10.1111/bjhp.12037. 23480457

[ref051] Misra-Hebert, A.D., Kay, R. and Stoller, J.K. (2004), “A review of physician turnover: rates, causes, and consequences”, American Journal of Medical Quality : The Official Journal of the American College of Medical Quality, Vol. 19 No. 2, pp. 56-66, doi: 10.1177/106286060401900203. 15115276

[ref052] Mitchell, T.R., Holtom, B.C., Lee, T.W., Sablynski, C.J. and Erez, M. (2001), “Why people stay: using job embeddedness to predict voluntary turnover”, Academy of Management Journal, Vol. 44 No. 6, pp. 1102-1121, doi: 10.2307/3069391.

[ref053] Mok, C., Boneham, W. and Lennon, M.J. (2020), “A ‘healthy’ health care workforce: insights into satisfaction and retention of doctors”, Medical Education, Vol. 54 No. 9, pp. 781-783, doi: 10.1111/medu.14269. 32557759

[ref088] Moreno-Jiménez, B., Gálvez-Herrer, M., Rodriguez, R. and Sanz Vergel, A.I. (2012), “A study of physicians’ intention to quit: the role of burnout, commitment and difficult doctor-patient interactions”, Psicothema, Vol. 24 No. 2, pp. 263-270. 22420355

[ref090] Morton, J. and Karen Schaab, P.H. (2008), “Factors influencing the departure of non-consultant hospital doctors from Christchurch, New Zealand”, The New Zealand Medical Journal, Vol. 121 No. 1273, pp. 13-24. 18480882

[ref087] Moss, P., Lambert, T., Goldacre, M. and Penelope, L. (2004), “Reasons for considering leaving UK medicine: questionnaire study of junior doctors’ comments”, BMJ, Vol. 329 No. 7477, p. 1263, doi: 10.1136/bmj.38247.594769.AE. 15469947 PMC534439

[ref092] Mousavi, S.M., Asayesh, H. and Sharififard, F. (2019), “Job satisfaction and turnover intention among anesthesiologists: an Iranian study”, Anesthesia and Pain Medicine, Vol. 9 No. 3, doi: 10.5812/aapm.83846. PMC6712281 31497515

[ref054] Ochsmann, E.B. (2012), “Thinking about giving up clinical practice? A gender-stratified approach to understanding junior doctors' choices”, Academic Medicine: Journal of the Association of American Medical Colleges, Vol. 87 No. 1, pp. 91-97, doi: 10.1097/acm.0b013e31823aba03. 22104056

[ref055] Oh, S. and Kim, H. (2019), “Turnover intention and its related factors of employed doctors in Korea”, International Journal of Environmental Research and Public Health, Vol. 16 No. 14, p. 2509, doi: 10.3390/ijerph16142509. 31337098 PMC6679070

[ref056] Ohly, S., Sonnentag, S., Niessen, C. and Zapf, D. (2010), “Diary studies in organizational research: an introduction and some practical recommendations”, Journal of Personnel Psychology, Vol. 9 No. 2, pp. 79-93, doi: 10.1027/1866-5888/a000009.

[ref057] Oliveri, R.S., Gluud, C. and Wille-Jørgensen, P.A. (2004), “Hospital doctors' self-rated skills in and use of evidence-based medicine – a questionnaire survey”, Journal of Evaluation in Clinical Practice, Vol. 10 No. 2, pp. 219-226, doi: 10.1111/j.1365-2753.2003.00477.x. 15189388

[ref091] Opoku, S. and Apenteng, B. (2014), “Seeking greener pastures? The relationship between career satisfaction and the intention to emigrate: a survey of Ghanaian physicians”, International Health, Vol. 6 No. 3, pp. 208-212, doi: 10.1093/inthealth/ihu030. 24958784

[ref058] Pantenburg, B., Kitze, K., Luppa, M., König, H.-H. and Riedel-Heller, S.G. (2018), “Physician emigration from Germany: insights from a survey in saxony, Germany”, BMC Health Services Research, Vol. 18 No. 1, p. 341, doi: 10.1186/s12913-018-3142-6. 29743052 PMC5944134

[ref059] Pappas, M.A., Stoller, J.K., Shaker, V., Houser, J., Misra-Hebert, A.D. and Rothberg, M.B. (2022), “Estimating the costs of physician turnover in hospital medicine”, Journal of Hospital Medicine, Vol. 17 No. 10, pp. 803-808, doi: 10.1002/jhm.12942. 35977052 PMC9547978

[ref060] Pathman, D.E., Konrad, T.R., Williams, E.S., Scheckler, W.E., Linzer, M. and Douglas, J. and Career Satisfaction Study Group. (2002), “Physician job satisfaction, dissatisfaction, and turnover”, Journal of Family Practice, Vol. 51 No. 7, pp. 593. 12160487

[ref061] Pfeffer, J. (2001), “What's wrong with management practices in Silicon Valley? A lot”, MIT Sloan Management Review, Vol. 42 No. 3, pp. 101-102.

[ref062] Pflipsen, J., McDermott, C., Doherty, E.M. and Humphries, N. (2019), “Why our doctors are leaving Irish emergency medicine training”, Irish Journal of Medical Science, Vol. 188 No. 4, pp. 397-1399, doi: 10.1007/s11845-019-01976-y. 30778849

[ref063] Poon, Y.S.R., Link, Y.P., Griffiths, P., Yong, K.K., Seah, B. and Liaw, S.Y. (2022), “A global overview of healthcare workers' turnover intention amid COVID-19 pandemic: a systematic review with future directions'”, Human Resources for Health, Vol. 20 No. 1, pp. 1-18, doi: 10.1186/s12960-022-00764-7. 36153534 PMC9509627

[ref064] Porter, C.M. and Rigby, J.R. (2021), “The turnover contagion process: an integrative review of theoretical and empirical research”, Journal of Organizational Behavior, Vol. 42 No. 2, pp. 212-228, doi: 10.1002/job.2483.

[ref065] Purl, J., Hall, K.E. and Griffeth, R.W. (2016), “A diagnostic methodology for discovering the reasons for employee turnover using shocks and events”, in Saridakis, G. and Cooper, C.L. (Eds), Research Handbook on Employee Turnover, Edward Elgar Publishing, pp. 213-246.

[ref066] Roy, A., van der Weijden, T. and de Vries, N. (2017), “Relationships of work characteristics to job satisfaction, turnover intention, and burnout amongst doctors in the district public-private mixed health system of Bangladesh”, BMC Health Services Research, Vol. 17, pp. 1-11, doi: 10.1186/s12913-017-2369-y. 28637454 PMC5480190

[ref067] Ruel, S. (2017), “Qualitative methods in business research”, Qualitative Research in Organizations and Management, Vol. 12 No. 1, pp. 87-88, doi: 10.1108/qrom-08-2016-1410.

[ref068] Sharma, A., Lambert, T.W. and Goldacre, M.J. (2012), “Why UK-trained doctors leave the UK: cross-sectional survey of doctors in New Zealand”, Journal of the Royal Society of Medicine, Vol. 105 No. 1, pp. 25-34, doi: 10.1258/jrsm.2011.110146. 22275495 PMC3265234

[ref069] Sherwood, R. and Bismark, M. (2020), “The ageing surgeon: a qualitative study of expert opinions on assuring performance and supporting safe career transitions amongst older surgeons”, BMJ Quality and Safety, Vol. 29 No. 2, pp. 113-121, doi: 10.1136/bmjqs-2019-009596. PMC7045790 31363015

[ref070] Smith, S.E., Tallentire, V.R., Pope, L.M., Laidlaw, A.H. and Morrison, J. (2018), “Foundation Year 2 doctors' reasons for leaving UK medicine: an in-depth analysis of decision-making using semistructured interviews”, BMJ Open, Vol. 8 No. 3, e019456, doi: 10.1136/bmjopen-2017-019456. PMC5855199 29500208

[ref071] Tsai, Y.-H., Huang, N., Chien, L.-Y., Chiang, J.-H. and Chiou, S.-T. (2016), “Work hours and turnover intention amongst hospital physicians in Taiwan: does income matter?”, BMC Health Services Research, Vol. 16 No. 1, p. 667, doi: 10.1186/s12913-016-1916-2. 27871296 PMC5117625

[ref072] Tziner, A., Rabenu, E., Radomski, R. and Belkin, A. (2015), “Work stress and turnover intentions amongst hospital physicians: the mediating role of burnout and work satisfaction”, Journal of Work and Organizational Psychology, Vol. 31 No. 3, pp. 207-213, doi: 10.1016/j.rpto.2015.05.001.

[ref073] Valle, M., Leupold, C.R. and Leupold, K.L. (2016), “Holding on and letting go: the relationship between job embeddedness and turnover amongst PEM physicians”, The Journal of Business Inquiry, Vol. 5 No. 1, pp. 3-10.

[ref074] Waldman, J.D., Kelly, F., Arora, S. and Smith, H.L. (2010), “The shocking cost of turnover in health care”, Health Care Management Review, Vol. 35 No. 3, pp. 206-211, doi: 10.1097/hmr.0b013e3181e3940e. 20551768

[ref075] Walsh, J. (2013), “Gender, the work-life interface and wellbeing: a study of hospital doctors”, Gender, Work and Organization, Vol. 20 No. 4, pp. 439-453, doi: 10.1111/j.1468-0432.2012.00593.x.

[ref076] Wang, J.-O., Li, C.Y., Kao, S., Yeh, T.C., Arens, J.F. and Ho, S.T. (2015), “Factors associated with Taiwan anesthesiologists' intention to leave anesthesia practice”, Journal of the Formosan Medical Association – Taiwan Yi Zhi, Vol. 114 No. 6, pp. 509-516, doi: 10.1016/j.jfma.2013.11.005. 24373937

[ref077] Williams, E.S., Konrad, T.R., Scheckler, W.E., Pathman, D.E., Linzer, M., McMurray, J.E., Gerrity, M. and Schwartz, M. (2010), “Understanding physicians' intentions to withdraw from practice: the role of job satisfaction, job stress, mental and physical health”, Health Care Management Review, Vol. 35 No. 2, pp. 105-115, doi: 10.1097/01.hmr.0000304509.58297.6f. 20234217

[ref078] Wilson, H.C.P., Abrams, S. and Simpkin Begin, A. (2021), “Drexit: understanding why junior doctors leave their training programs to train overseas – an observational study of UK physicians”, Health Science Reports, Vol. 4 No. 4, pp. e419-n/a, doi: 10.1002/hsr2.419. 34646946 PMC8499680

[ref079] World Health Organization (WHO) (2022), “Global strategy on human resources for health: workforce 2030 – reporting at seventy-fifth World Health Assembly”, World Health Organization, available at: https://www.who.int/news/item/02-06-2022-global-strategy-on-human-resources-for-health--workforce-2030

[ref080] Wu, Y.-F., Wang, P.-C. and Chen, Y.-C. (2018), “Gender differences and work-family conflicts amongst emergency physicians with intention to leave”, Emergency Medicine International, pp. 1-9, doi: 10.1155/2018/3919147. PMC6231391 30510802

[ref081] Zhang, Y. and Feng, X. (2011), “The relationship between job satisfaction, burnout, and turnover intention amongst physicians from urban state-owned medical institutions in Hubei, China: a cross-sectional study”, BMC Health Services Research, Vol. 11 No. 1, p. 235, doi: 10.1186/1472-6963-11-235. 21943042 PMC3197494

[ref082] Zhang, F., Luo, Z., Chen, T., Min, R. and Fang, P. (2017), “Factors affecting turnover intentions amongst public hospital doctors in a middle-level city in central China”, Australian Health Review, Vol. 41 No. 2, pp. 214-221, doi: 10.1071/ah15238. 27120079

[ref083] Zhang, C., Hu, L., MaWu, J.S., Guo, J. and Liu, Y. (2019), “Factors determining intention to leave amongst physicians in tertiary hospitals in China: a national cross-sectional study”, BMJ Open, Vol. 9 No. 3, e023756, doi: 10.1136/bmjopen-2018-023756. PMC6429748 30872540

